# The accuracy of placental alpha-microglobuline-1 test in diagnosis of premature rupture of the membranes 

**Published:** 2015-06

**Authors:** Maryam Khooshideh, Vida Radi, Reihaneh Hosseini, Ladan Hosseini

**Affiliations:** 1 *Department of Obstetrics and Gynecology, Arash Hospital, Tehran University of Medical Sciences, Tehran, Iran.*; 2 *Research Development Center, Arash Hospital, Tehran University of Medical Sciences, Tehran, Iran.*

**Keywords:** *Placental alpha microglobulin-1*, *Premature ruptures of membrane*, *Diagnostic test*

## Abstract

**Background:**

Premature rupture of membranes (PROM) is a common obstetric issue during pregnancy which might lead to serious fetal or maternal problems. Therefore, an appropriate diagnosis and management of PROM are of significant importance in patients.

**Objective:**

The aim of this study was to determine the accuracy of placental alpha microglobuline-1 (PAMG-1) test in PROM diagnosis and compare this diagnostic method with other standard tests in diagnosis of PROM.

**Materials and Methods:**

In this prospective diagnostic accuracy study, patients with symptoms of membrane rupture in 16-39 weeks of gestation were involved. Three tests including Fern, Nitrazine and PAMG-1 were performed at the same time.

**Results:**

PROM was confirmed in 86 patients out of 100. The sensitivity and specificity were respectively 81.3% and 100% for Fern test, 93% and 92.8% for Nitrazine test, 98.9% and 92.8% for PAMG-1 test. PAMG-1 test showed higher sensitivity (98.9% with p<0.001) and accuracy (98%) compared with conventional tests. Although PAMG-1showed a lower positive predictive value (PPV) compared to conventional tests such as Fern test (100%), it was shown to be more accurate.

**Conclusion:**

The accuracy of PAMG-1 test was superior to both Fern and Nitrazine test in PROM diagnosis.

## Introduction

Premature rupture of membranes (PROM) is known as one of the most common problems during pregnancy and occurs when the membrane of amniotic sac and chorion ruptures more than one hour before the beginning of labor. It occurs in 10% of pregnancies (1). PROM could lead to either maternal or fetal hurts such as infections (neonatal sepsis or chorioamnionitis), preterm delivery or stillbirth. PROM is sometimes difficult to diagnose. The clinicians should evaluate the risk of maintaining the pregnancy against serious consequences. A correct and precise diagnostic method would help in patient management. On the other hand, false positive diagnosis could lead to improper measures and unnecessary labor interventions.

Conventional methods for PROM diagnosis besides pooling test are Fern and Nitrazine tests. Previous studies have investigated the accuracy of these tests. Based on a study conducted by Haan *et al* Fern test predicted the rupture of membranes correctly in 63% and incorrectly in 29% of patients and the test was more accurate in patients of labor phase (2). In a review article by Gallot *et al* Nitrazine test in vaginal pH assessment had sensitivity of 73-91% and specificity of 72-83% (3). The validity of these tests largely depends on examiner`s skill in performing the tests and reporting the results. However, there are some conditions leading to false positive or false negative results. Additionally, conditional tests need applying speculum which is unpleasant for patients. There have been several efforts on finding easier and more accurate tests for PROM diagnosis.

The tests are based on the detection of insulin- like growth factor binding protein-1 (IGFBP-1), alpha fetoprotein (AFP), β-hCG or Placental Alpha Microglubolin I (PAMG-1) (4, 5). PAMG-1 is released from decidual tissue (6). Measurement of Placental Alpha Microglubolin I (PAMG-1) is one of the newest tests. It is a rapid and easy test performed within 5-10 min. Speculum is not used in this test. Some investigators have claimed that PAMG-1 test is more accurate than conventional diagnostic methods (6, 7). Alkaline vagina and presence of blood have no effect on the result of the test (8). IGBP-1 is a protein in amniotic fluid which has been used for PROM detection. Tagore *et al* have reported that PAMG-1 test was more accurate than IGFBP-1 but Abdelazim *et al* announced no significant difference between these two tests (8, 9).

Also, there are some studies about β-hCG measurement in vaginal fluid but the reported accuracy is lower than PAMG-1 (10, 11). The aim of this study was to investigate the sensitivity, specificity, negative or positive predictive value of PAMG-1 diagnostic test and evaluate it as a gold standard in PROM diagnosis. In previous studies PAMG-1 test was compared simply with Fern or Nitrazin tests but these recent tests do not have 100% accuracy as standard tests.

In this study we followed patients and repeated the tests in some cases in order to detect false negative cases and improve accuracy evaluation.

## Materials and methods

This prospective diagnostic accuracy study was approved by the Ethics Committee of Tehran University of Medical Sciences. The informed consent was obtained from all participants.

In total, 100 pregnant women with gestational age of 16-39 weeks with the complaint of leakage during 24 hr prior to coming to the hospital admitted to Arash Women’s Hospital within March 2011 to September 2013 were included in the study. Patients with vaginal bleeding or signs of chorioamnionitis were excluded from the study. All with the complaint of leakage were at first investigated through complete medical history and clinical examination without using disinfectants.

Clinical examination consisted of observing clear flow from the cervix spontaneously or after coughing or observing filling of amniotic fluid in Posterior Fornix (pooling test). Three tests were performed at the same time: Fern, Nitrazine and PAMG-1. Then the results of all three tests were compared to each other and the proven presence of PROM. For ferning test we avoided to use any lubricants or antiseptics, used a sterile swab for preparing a thin smear on a glass microscope slide by spreading evenly, allowed the slide to air dry and examined the fully-dried slide microscopically, using the 10× objective. Observe for "fern-like" crystals. Presence of branching view of crystals indicates that the fluid is amniotic fluid. All fern test were evaluated by senior resident who was a member of research team.

In Nitrazine test we applied patient sample from swab onto the strip of Nitrazine paper. Immediately we matched the color on the strip with the closest color on the dispenser color chart. If the pH paper color is yellow to olive green, the corresponding pHis 4.5-6.0. The test is negative for amniotic fluid. If the pH paper color is blue-green to deep blue, the corresponding pHis 6.5-7.5. The test is positive for amniotic fluid. PAMG-1 was tested using AmniSure in all patients. Immunochromatography kit is one-step test based on specific monoclonal antibodies which have the ability to reveal amounts of protein in amniotic fluid (PAMG-1). The protein is secreted from decidual part of placenta. The AmniSure ROM test (placental α-microglobulin-1 immunoassay, AmniSure ROM test, International LLC, Boston, Ma, USA) has a sensitivity threshold of 5 ng per ml.

The sample of this test was collected from the secretions of vagina. A sterile polyester swab provided by the manufacturer was inserted into vaginal fornix for one min. The head of the swab was shaken for a minute in vial containing solvent. After removing the swab, the test strip was dipped into the solvent and the result was determined after 5-10 min. This immunoassay test was reported as negative or positive. In this test, the emergence of 1 line and 2 lines indicated a negative and a positive result for PROM respectively.

If no line emerges, the test needs to be replicated. Patients showing negative results with both conventional and AmniSure tests were considered as negative in terms of leakage, but they were hospitalized for further observations. The women showing negative results with one test and positive results with the other one were also hospitalized and examined during the hospitalization in terms of leakage of cervical fluid. The interrupted pattern of the leakage in some patients led us to repeat the conventional tests 1-3 days after admission to hospital.

The final diagnosis was made according to the patients’ observation more than 5 days, sonography or progress to delivery. The cases that were diagnosed as PROM after admission and their primary test results were negative, were considered as false negative for that test. At the time of admission, patients diagnosed with proven PROM, with gestational age below 23 weeks and obvious continuous fluid leakage were managed to terminate the pregnancy. Furthermore, patients at the 23^rd^-34^th^ weeks of gestational age were determined to receive conservative managements and patients above this age were determined to terminate the pregnancy.

Symptoms such as unexplained tachycardia, predominant leukocytosis or fever with a history of leakage were considered as probable chorioamnionitis and indications to terminate the pregnancy. Patients underwent conservative management were controlled in terms of vital signs and temperature every 6 hr. Complete blood count (CBC), erythrocyte sedimentation rate (ESR) and C-reactive protein (CPR) were also measured twice a week. Antibiotic prophylaxis was prescribed for one week and glucocorticoids were used after 27 weeks of pregnancy.

**Statistical analysis**

The sensitivity, specificity, positive predictive value (PPV), and negative predictive value (NPV) of AmniSure test in diagnosis of PROM were compared with other conventional tests. Data analysis was performed using SPSS (Statistical Package for the Social Sciences, version 17.0; IBM, Armonk, NY, USA). Numerical variables were presented as mean±SD, and categorical variables were presented as number and percentage. Sensitivity, specificity, positive predictive value (PPV) and negative predictive value (NPV) of each test were calculated. Accuracy is the proportion of the true results of the test in population. Mc Nemar test was used to compare the sensitivity and specificity between tests in predicting PROM. P<0.05 was considered statistically significant. Using data of previous studies, setting the type-1 error (a) at 0.05, the power at 0.8, the sample size of this study was calculated 100 women (3).

## Results

**Figure 1:**
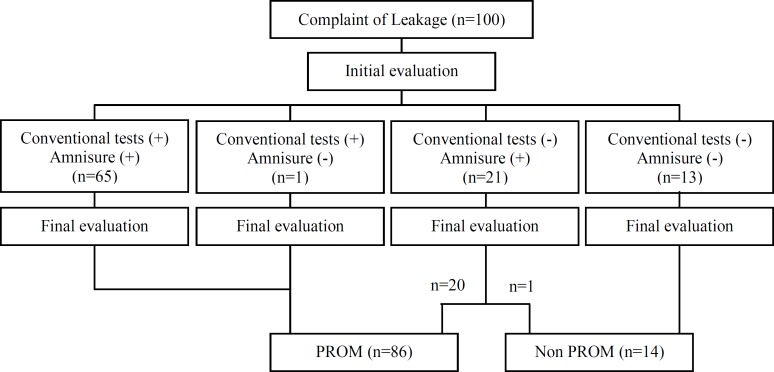
Diagram of patients. Conventional tests (+): Fern test and/ or Nitrazin test are positive. Conventional tests (-): Fern Test and Nitrazin test are negative

The average age of 100 pregnant women at the 16^th^-39^th^ weeks of gestation involved in the study was 26±5.5 years (17-40 years old) and average of gestational age was 34.2±6.2 weeks. 55% of pregnancies were term, 19% between 34-37 weeks and 26% less than 34 weeks. The results of tests have been shown in Figure 1. Initial observations and conventional tests confirmed PROM in 66 women and PAMG-1 test indicated PROM in 86 women. Finally 86% of involved mothers were diagnosed with PROM. Among 21 cases which were negative in at least one conventional test and positive in PAMG-1 test, 20 cases had PROM. Among these 20 cases, 14 cases had positive Nitrazine test and 5 cases had positive Ferning test.

In these patients the obvious signs of PROM emerged averagely within 9 hr (in 16 cases within 24 hr, in 3 cases within 2 days, and in 1 case within 7 days). One case showed false positive result of PAMG-1, while the conventional tests were negative for her. The case was a woman with 18 weeks of gestational age who observed leakage after cervical cerclage. PAMG-1 test was positive, but conventional tests were negative for her. After a week the results of all tests changed to be negative. A false negative case was a 24-week patient whose Nitrazine test was positive and PAMG-1 and Ferning tests were negative. After a week, labor started spontaneously.

PAMG-1 test has 98.9% sensitivity, 92.8% specificity, 99% PPV and 93% NPV in ROM diagnosis. The sensitivity, specificity, PPV and NPV of Nitrazine test were 93%, 92.8%, 98.7% and 68.4% respectively, whereas, Fern test has 81.3% sensitivity, 100% specificity, 100% PPV and 46.6% NPV. PAMG-1 test has also higher sensitivity than Fern test (p=0.00) but in terms of specificity there was no significant difference, although, the specificity of Fern was more than PAMG-1. The sensitivity of PAMG-1 test did not noticeably differ from Nitrazine test (98.8% vs. 93%, p=0.14). The accuracy of the applied tests was 98%, 93%, 84% respectively for PAMG-1, Nitrazine and Fern tests which is higher in PAMG-1 test.

**Table I:** Performance metric of all tests

	**SN**	**SP**	**PPV**	**NPV**	**Accuracy**	**False positive rate**	**False negative rate**
Amnisure test	98.8%	92.8%	99%	93%	98%	7%	1%
Pooling test	76.7%	100%	100%	41%	80%		
Nitrazin test	93%	92.8%	98.7%	68.4%	93%		
Fern test	81.3%	100%	100%	46.6%	84%		

SN: sensitivity

SP: specificity

PPV: positive predictive value

NPV: negative predictive value

**Table II:** Comparison between tests (Mc Nemar Test)

	**Amnisure and fern**	**Amnisure and nitrazin**	**Amnisure and pooling test**
SN (p-value)	<0.001	0.062	<0.001
SP (p-value)	1.000	1.000	1.000

SN: sensitivity

SP: specificity

## Discussion

Since PROM and its serious consequences are known as common obstetric problems, finding a sensitive, specific, non-invasive and rapid test for its diagnosis is of great important. Such test will be able to be a gold standard for diagnosis of PROM. Placental alpha microglobulin-1 (PAMG-1) diagnostic test is an immunochromatography method performed in one step in which some monoclonal antibodies are used to detect the PAMG-1 proteins. PMAG-1 is a 34 kilo Dalton protein found in amniotic fluid. There is a high value of this protein in amniotic fluid but it is rarely found in blood and vaginal secretions. Almost 1000-10,000 times difference between the concentration of amniotic fluid and cervical secretions makes PAMG-1 a suitable marker in ROM diagnosis (12-14). Minimum threshold diagnostic AmniSure is about 5 ng/ml.

The sensitivity of the test for PROM diagnosis at this concentration was estimated to be 99%. In case of bacterial vaginosis or presence of blood, the level of PAMG-1 will not be more than 3 ng/mL in vaginal secretions which is lower than the threshold sensitivity of this test (5 ng/ml) (7). In the present study, the sensitivity and NPV of PAMG-1 test were certainly higher than conventional tests. In addition, according to Abdelazim *et al* study in pregnancies more than 37 weeks, the sensitivity and specificity of PAMG-1 in PROM diagnosis were 97.33% and 98.67%, while the sensitivity and specificity were respectively reported as 84% and 78.67% for Fern test and 86.67% and 81.33% for Nitrazine test (15).

Indeed, positive result of PAMG-1 test approved ROM in many cases whose conventional tests were negative. It seems that the main advantage of PAMG-1 is its higher sensitivity and NPV in comparison with other tests which indicate the low threshold of test in detecting PROM. In another study conducted by Cousins *et al* which had the same design as ours on 203 pregnant women with gestational age of 15-42 weeks, AmniSure test had sensitivity of 98.9%, specificity of 100%, PPV of 100% and NPV 99.1% which were in line with our results (12). More similar results have been shown in several studies (6, 8, 16-18).

According to Hann’s study, the result of Fern test predicted the actual status of membranes correctly in 63% and incorrectly in 29% of patients without labor pain (2). Gallot *et al* also reported 73-91% sensitivity and 72-83% specificity for Nitrazine test (3). In the present study, Fern test had the highest specificity and PPV, but the weak point of it was NPV, so, its false negative rate is high. False positive results might occur in specimens contaminated with semen, urine, or cervical mucus (19). False negative results may occur due to prolonged rupture of the membranes (longer than 24 hr), blood or heavy discharge. Furthermore, false negative results may occur if only a small volume of fluid has leaked (20). Moreover, Nitrazine test showed lower sensitivity and NPV compared to PAMG-1 test. In Friedman’s study a false negative rate of 12.9% was observed in Nitrazine test (19). In their study the false positive rate was 17.4%, while in our study this rate was lower. It could be due to omitting disinfectant in examination or the exclusion of patients with severe bleeding. As Haan and Friedman have reported false positive result arises from cervicitis, vaginitis, alkaline urine, blood, semen and antiseptics (2, 19).

Since Nitrazine test is close to PAMG-1 test in terms of accuracy, SP, SN, and PPV, it can be used in cases when PAMG-1 is not accessible or in patients who are not able to afford the test expense. Traditional Nitrazine test can be used in cases that there is not blood or bacterial vaginosis. In PAMG-1 test the rate of false negative result is significantly low. Marcellin *et al* in 80 pregnancies with or without PROM compared the diagnosis of PROM using PAMG-1 vs. IGFBP-1 in cervico-vaginal secretions. In this study, accuracy of both tests for diagnosis of PROM was the same. The sensitivity of AmniSure was also 95% and its specificity was 94.8% in this study (21). Measurement of IGFBP-1 is also another technique which is under investigation.

The advantages of PAMG-1 test are that it is a rapid and easy test performed within 5-10 min. It could be used without speculum and it is claimed that this test it is more accurate than conventional accessible methods. Alkaline vagina and presence of blood has no effect on the result of the test. The high cost of the test is its weak point but the more precise diagnosis would save resources at the end.

**Limitation**

One limitation of our study was the low portion of patients without PROM. Indeed, the low positive results indicate the highly selective patients. Also, we did not have a real gold standard method for evaluating accuracy. Therefore, because of the absence of this gold standard, we considered the final clinical diagnosis as the gold standard. A case- control study will be useful for further evaluation of tests. Our study was performed on patients suspected as PROM cases; therefore further case-control studies might complete our findings. Evaluation of other biochemical markers and an improvement in PAMG-1 test are recommended in order to achieve an accurate test as the gold standard in PROM diagnosis, although PAMG-1 test seems to be a fine choice today.

## Conclusion

PAMG-1 is a test with high accuracy and it can be suggested as a replacement for conventional tests in equivocal cases.
